# Crystallization of colourless hexanitratoneptunate(iv) with anhydrous H^+^ countercations trapped in a hydrogen bonded polymer with diamide linkers†

**DOI:** 10.1039/c9ra10090c

**Published:** 2020-02-07

**Authors:** Koichiro Takao, Juliane März, Moe Matsuoka, Takanori Mashita, Hiroyuki Kazama, Satoru Tsushima

**Affiliations:** Laboratory for Advanced Nuclear Energy, Institute of Innovative Research, Tokyo Institute of Technology 2-12-1 N1-32, O-okayama, Meguro-ku 152-8550 Tokyo Japan ktakao@lane.iir.titech.ac.jp; Institute of Resource Ecology, Helmholtz-Zentrum Dresden-Rossendorf (HZDR) Bautzner Landstraße 400 01328 Dresden Germany j.maerz@hzdr.de s.tsushima@hzdr.de; Tokyo Tech World Research Hub Initiative (WRHI), Institute of Innovative Research, Tokyo Institute of Technology 2-12-1, O-okayama, Meguro-ku 152-8550 Tokyo Japan tsushima.s.ab@m.titech.ac.jp

## Abstract

Colourless crystalline compounds of centrosymmetric [Np(NO_3_)_6_]^2−^ were yielded from 3 M HNO_3_ aq in the presence of double-headed 2-pyrrolidone derivatives (L). In the obtained crystal structures, H^+^ was also involved as a countercation to compensate for the negative charge of [Np(NO_3_)_6_]^2−^, where the initial hydration around H^+^ was fully removed during crystallization despite it having the strongest hydration enthalpy. Instead, this anhydrous H^+^ was captured by L to form a [H^+^⋯L]_*n*_ hydrogen bonded polymer. In [Np(NO_3_)_6_]^2−^, the Np^4+^ centre is twelve-coordinated with 6 bidentate NO_3_^−^, and therefore, present in an icosahedral geometry bearing inversion centre. In such a centrosymmetric system, any f–f transitions stemming from the 5f^3^ electronic configuration of Np^4+^ are electric-dipole forbidden. This is the reason why the compounds currently obtained were colourless unlike ordinary Np(iv) species, which are olive-green.

## Abstract 2

Centrosymmetric hexanitratoneptunate(iv) crystallizes with anhydrous proton countercations trapped in a hydrogen bonded polymer with diamide linker molecules to give colourless crystals.

## Graphical abstract

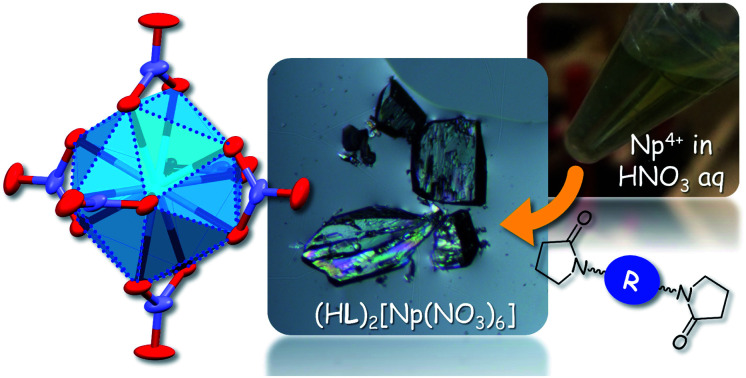

## Introduction

The coordination chemistry of actinides is highly relevant to nuclear chemical engineering, especially for reprocessing of spent nuclear fuels and geological disposal of radioactive wastes.^1^ Aqueous HNO_3_ is most popularly employed to dissolve the spent nuclear fuels and to separate U and Pu from fission products and minor actinides with solvent extraction. Therefore, actinide coordination chemistry of nitrato complexes is the most essential to systematically understand the separation behaviour of actinides.^2^ Among the various oxidation numbers available for actinide elements relevant to nuclear fuel recycling, the tetravalent state, An(iv), is highly important especially in terms of Pu^4+^ separation by solvent extraction with tri-*n*-butyl phosphate (TBP). The limiting An^4+^ complexes with NO_3_^−^ are [An(NO_3_)_6_]^2−^ (An = Th,^3–7^ U,^8,9^ Np,^10,11^ Pu^12–14^), which usually crystallize with various countercations having relatively low hydration enthalpy like heavy alkali metals and quaternary ammonium ions (NR_4_^+^, R = H, alkyl).^15^ To our best knowledge, all the reported compounds of An(iv) usually exhibit original colours defined by their respective 5f electronic configurations.

**Fig. 1:**
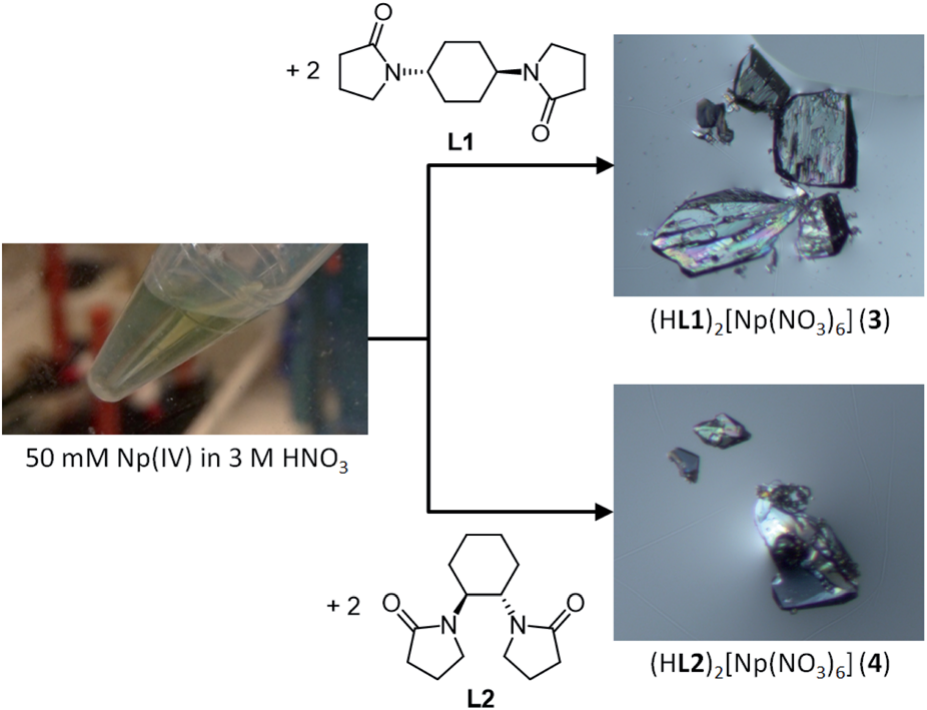
Reaction scheme to prepare colourless crystalline compounds, (HL1)_2_[Np(NO_3_)_6_] (3) and (HL2)[Np(NO_3_)_6_] (4) from Np^4+^ with L1 and L2 in 3 M HNO_3_ aq. An (*S*,*S*)-isomer of L2 is only shown here, whereas its racemate has been used in this work.

In contrast, we recently demonstrated that H^+^ showing the highest hydration enthalpy^3^ also has a potential to make crystalline compounds with [U(NO_3_)_6_]^2−^ and NO_3_^−^ by coupling with diamide linker molecules appropriately selected to allow strong hydrogen bond showing little potential barrier along atomic coordinate of H^+^ between hydrogen bond donor and acceptor, where we employed double-headed 2-pyrrolidone derivatives like L1 and L2 shown in Fig. 1.^16,17^ The resulting U(iv) compounds have a general formula of (HL)_2_[U(NO_3_)_6_] (L = L1 (1), L2 (2)). In these crystal structures, H^+^ countercations are fully dehydrated despite deposition from the aqueous HNO_3_ solutions. Instead, these anhydrous H^+^ are trapped into a cavity between two carbonyl O atoms of the neighbouring L molecules to form a unique hydrogen bond polymer, [H^+^⋯L]_*n*_. Interestingly, 1 and 2 do not exhibit characteristic green colour of U(iv), but are nearly colourless. Thanks to separation of U^4+^ centre from H^+^ (>5.8 Å), U^4+^ in these compounds are located in icosahedral geometries with nearly perfect *T*_h_-symmetry. In such a centrosymmetric system, the f–f transitions stemming from its 5f^2^ electronic configuration becomes electric-dipole (*i.e.*, Laporte) forbidden, making 1 and 2 nearly colourless. This assumption was further corroborated by complete active space self-consistent field (CASSCF) calculation including spin–orbit coupling.

To our understanding, crystallization of [U(NO_3_)_6_]^2−^ with anhydrous H^+^ is quite exceptional from usual trend of [An(NO_3_)_6_]^2−^. Furthermore, formation of nearly colourless U(iv) is also unprecedented because An(iv) usually exhibit characteristic colour due to visible-light absorption arising from its own f–f transitions. There remains open questions whether crystallization of [An(NO_3_)_6_]^2−^ as anhydrous H^+^ salts like 1 and 2 is peculiar to U^4+^ or common in An(iv), and whether 5f orbitals remain separated enough from those of NO_3_^−^ to still make [An(NO_3_)_6_]^2−^ colourless. To answer these points, we decided to employ Np^4+^ as another An^4+^. In this paper, we report details about synthesis and structural characterization of [Np(NO_3_)_6_]^2−^ crystallized together with H^+^ in aid of L1 (3) and L2 (4).

## Experimental

### Materials

**Caution**! ^237^Np is a radioactive isotope (specific activity: 2.63 × 10^7^ Bq g^−1^ with *T*_1/2_ = 2.14 × 10^6^ years) and an alpha emitter. It has to be handled in dedicated facilities with appropriate equipment for radioactive materials to avoid health risks caused by radiation exposure.

All the operations to handle Np have been done in a dedicated glove box in the control area of HZDR. Reaction scheme is shown in Fig. 1. A stock solution of Np^4+^ (50 mM) was prepared by dissolving NpCl_4_(DME)_2_ (13.9 mg)^18^ in 3 M HNO_3_ aq. The concentration of Np^4+^ in this stock solution was determined by γ-ray spectrometry. The diamide linker molecules (L1 and L2) were prepared through a method reported elsewhere.^19–21^ The Np^4+^ stock solution (50 mM, 50 μL) was layered with 3 M HNO_3_ aq (30 μL) and 3 M HNO_3_ solution of 0.50 M diamide (L1 or L2, 10 μL) in a *ϕ*5 mm glass test tube. Slow diffusion of Np^4+^ and L resulted in deposition of colourless crystals of (HL)_2_[Np(NO_3_)_6_] (L = L1 (3), L2 (4)) in 88% and 91% yield, respectively.

### Methods

The deposited crystals were covered with mineral oil, followed by being mounted on a MicroMount. Single crystal X-ray diffraction patterns were recorded by D8 VENTURE diffractometer (Bruker) with micro-focused Mo Kα radiation (*λ* = 0.71073 Å). The frames were integrated with the Bruker SAINT software package using a narrow-frame algorithm. Absorption correction by SADABS^22^ was applied which resulted in transmission factors described in the crystallographic information file of each compound. The obtained data were processed by Olex2.1.2 software package^23^ suited with SHELX program.^24^ The structures of 3 and 4 were solved by direct method, SHELXS,^25^ and expanded using Fourier techniques. All non-hydrogen atoms were anisotropically refined by SHELXL-2017/1.^24^ Anhydrous H^+^ in each compound were isotropically refined, whereas all other hydrogen atoms were refined using the riding model. The final cycle of full-matrix least-squares refinement on *F*^2^ was based on observed reflections and parameters, and converged with unweighted and weighted agreement factors, *R* and *w*R, respectively. Crystallographic data of 3 and 4 were summarized in Table 1, and compared with those of U(iv) analogues, (HL)_2_[U(NO_3_)_6_] (L = L1 (1), L2 (2)) we reported previously^16^ in Tables S1 and S2 (ESI†), respectively.

**Table 1:** Crystallographic data of (HL)_2_[Np(NO_3_)_6_] (L = L1, L2)

	(HL1)_2_[Np(NO_3_)_6_], 3	(HL2)_2_[Np(NO_3_)_6_], 4
Formula	C_28_H_46_N_10_NpO_22_	C_28_H_46_N_10_NpO_22_
fw	1113.76	1113.76
Crystal size (mm)	0.075 × 0.098 × 0.157	0.080 × 0.098 × 0.110
Cryst. system	Monoclinic	Monoclinic
Space group	*C*2/*c* (#15)	*P*2_1_/*n* (#14)
*a* (Å)	18.1413 (10)	9.8264 (10)
*b* (Å)	10.9944 (6)	10.7164 (11)
*c* (Å)	21.6888 (12)	19.517 (2)
*β* (°)	109.931 (2)	103.175 (4)
*V* (Å^3^)	4066.8 (4)	2001.1 (4)
*Z*	4	2
*T* (K)	100	100
*D*_calcd_ (g cm^−3^)	1.814	1.848
μ (mm^−1^)	2.652	2.695
Obsd data (all)	4312	4249
*R* (*I* > 2*σ*)	0.0192	0.0188
w*R* (all)	0.0420	0.0691
GOF	1.071	1.340
Δ*ρ*_max_ (e^−^ Å^−3^)	0.474	0.975
Δ*ρ*_min_ (e^−^ Å^−3^)	−0.523	−1.196

The γ-ray analysis of ^237^Np in the stock solution and supernatants after the deposition of 3 and 4 have been performed at VKTA Dresden. Each solution sample (0.5 or 1 μL) was loaded into a *ϕ*5 mm glass test tube, followed by loading to the γ-ray detector. The γ-ray emission from the sample was counted for 20 min real time plus 0.05% dead time.

For colour component analysis, the photomicrographs of Np(iv) crystalline compounds shown in Fig. 1 were processed by ImageJ (ver. 1.52a)^26^ to extract colour appearance parameters, hue, saturation, and brightness in 8 bit grey scale.

### Theoretical calculations

Electronic absorption spectra of Np(iv) complexes were calculated at the CASSCF (3,7) level using ORCA program version 4.0.^27^ The coordinates of [Np(NO_3_)_6_]^2−^ were taken from the crystal structures of 3, while those of aqua species [Np(H_2_O)_*n*_]^4+^ (*n* = 8, 9)^28^ were preliminarily optimized by DFT calculations (Gaussian 16 B.01)^29^ at the B3LYP level in water. Energies and wavefunctions were calculated by the CASSCF/sc-NEVPT2 (strongly contracted *n*-electron valence state perturbation theory) approach. CASSCF calculations using ORCA were performed following the protocol provided by Prof. Frank Neese and his coworkers.^30,31^ An active space considering the seven 5f orbitals was employed in the calculations whereas the number of roots was set to include all possible states stemming from 5f^3^ configuration of both doublet and quartet states. For Np, segmented all-electron relativistically-contracted basis sets of valence triple-zeta quality with polarization functions adapted to the Douglas–Kroll–Hess Hamiltonian (SARC-DKH-TZVP) were used. For all other atoms, scalar relativistically recontracted Karlsruhe valence triple-zeta basis sets were employed. Spin–orbit coupling (SOC) effects are included by quasi-degenerate perturbation theory (QDPT), where the multiplets stemming from the *S* = *M*_s_ CASSCF states are mixed by the spin–orbit mean field (SOMF) operator. Calculated spectra do not include vibronic progression to the electronic transitions.

## Results and discussion

In accordance with reaction scheme shown in Fig. 1, Np^4+^ reacted with L1 and L2 in 3 M HNO_3_ aq. In a glass test tube (∼5 mm O.D.), this Np(iv) stock solution (50 μL, Np^4+^: 2.5 μmol) was carefully layered with 3 M HNO_3_ aq (30 μL), and 3 M HNO_3_ solution of L (0.50 M, 10 μL, L: 5.0 μmol). These reaction mixtures were stored at silent place in an Np-dedicated glove box overnight to allow slow diffusion of Np^4+^ and L. As a result, crystalline compounds 3 and 4 grew up from the reaction mixtures of L1 and L2, respectively. Interestingly, the characteristic olive colour of Np(iv) (see Fig. 1) remarkably faded upon crystal growth, while the deposited compounds were also colourless. According to γ-ray spectrometry, the Np(iv) stock solution contained 315 kBq mL^−1^ of ^237^Np, whereas only 20.6 kBq mL^−1^ and 15.2 kBq mL^−1^ were found in the supernatants of the L1 and L2 systems after crystal deposition, respectively. Thus, Np precipitated as colourless crystals 3 and 4 from the reaction mixtures in 88% and 91% yield, respectively. Due to strong radioactivity of ^237^Np (2.63 × 10^7^ Bq g^−1^), further characterization methods like elemental analysis, IR, and powder XRD are unavailable at present. However, taking into account our recent results of well-characterized U(iv)-analogues, (HL)_2_[U(NO_3_)_6_] (L = L1 (1), L2 (2)),^16^ the most probable identities of 3 and 4 are crystalline salts of a centrosymmetric hexanitratoneptunate(iv), [Np(NO_3_)_6_]^2−^.

**Fig. 2:**
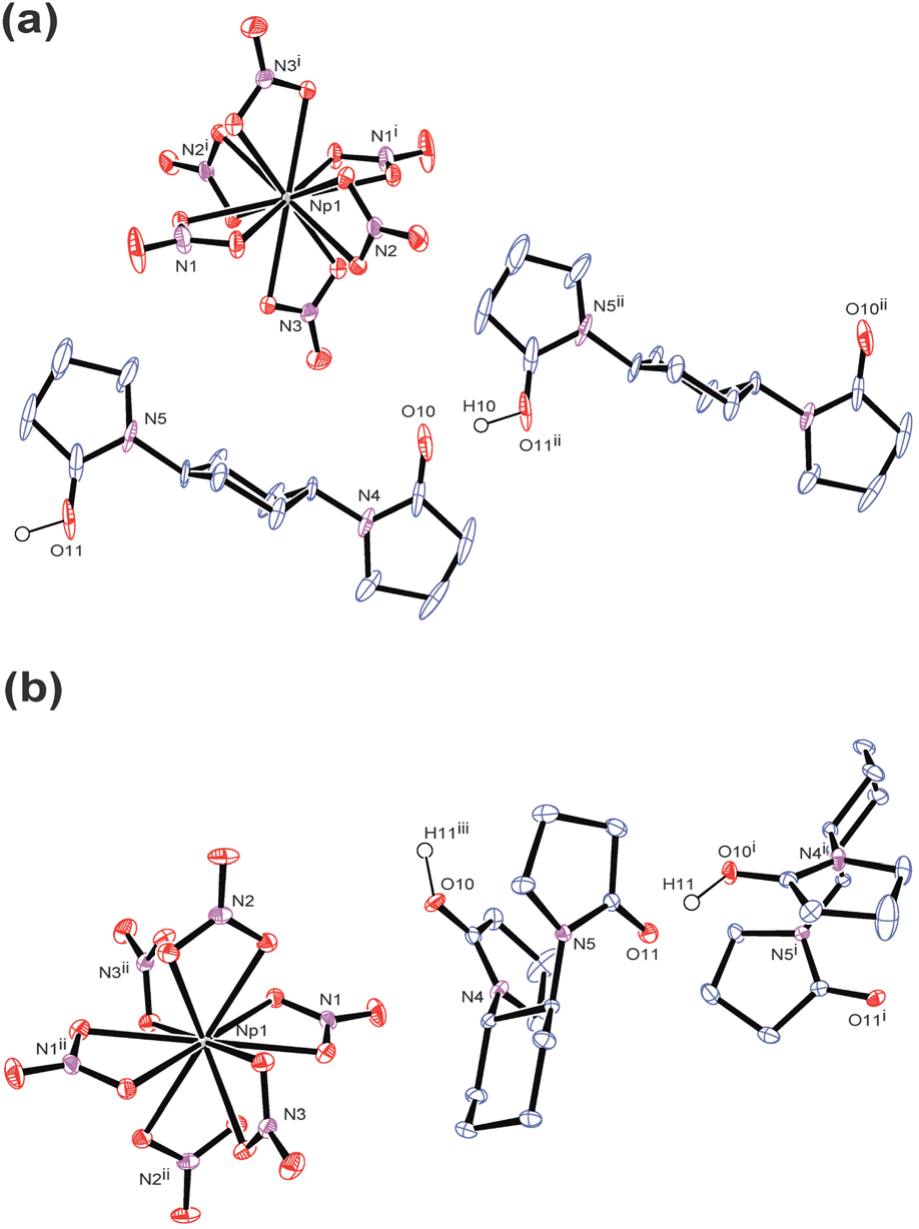
ORTEP views of (a) (HL1)_2_[Np(NO_3_)_6_] (3) and (b) (HL2)[Np(NO_3_)_6_] (4) at 50% probability level. Each one of disordering atoms of L1 and H atoms except for H^+^ involved in the hydrogen bond polymers are omitted for clarity. Both (*R*,*R*)- and (*S*,*S*)-enantiomers of L2 are present in the crystal structure of 4 to make it racemic, while only the (*S*,*S*)-isomer is displayed here.

Indeed, the single crystal X-ray analysis resulted in the precise molecular and crystal structures of (HL)_2_[Np(NO_3_)_6_] (L = L1 (3), L2 (4)) as shown in Fig. 2. As predicted from the U(iv)-analogues (1, 2), these compounds consist of [Np(NO_3_)_6_]^2−^ together with two L molecules. As shown in Tables S1 and S2 (ESI†), lattice parameters of 3 and 4 are almost identical with those of the corresponding U(iv)-analogues 1 and 2,^16^ respectively, indicating that the obtained Np compounds are isomorphic and isostructural to these U(iv)-analogues. These facts are strong evidence that the Np centre in both 3 and 4 still remains tetravalent. The selected structural parameters of 3 and 4 are summarized in Table 2 together with those of the U(iv)-analogues, 1 and 2.^16^ Bidentate manner of NO_3_^−^ resulted in dodeca-coordination around the Np^4+^ centre to make an icosahedral geometry. Interatomic distances between Np and O of NO_3_^−^ are 2.49–2.51 Å in 3 and 2.48–2.52 Å in 4. These bond lengths seem to be nearly the same with those in bipyridinium dication salts of [Np(NO_3_)_6_]^2−^ (2.48–2.53 Å, 2.48–2.54 Å),^10,11^ while tend to be slightly shorter than those found in [U(NO_3_)_6_]^2−^ of 1 (2.51–2.53 Å) and 2 (2.50–2.53 Å), respectively.^16^ The latter contrast can be ascribed to a typical trend of the actinide contraction.^2^ Considering similarity in chemical behaviour frequently observed in the actinide series, formation of isostructural compounds of Np with U is quite reasonable.

**Table 2:** Selected structural parameters of (HL)_2_[An(NO_3_)_6_] (An = U, Np; L = L1, L2)

	1^a^	2^a^	3^b^	4^b^
An=	U	U	Np	Np
L=	L1	L2	L1	L2
**Bond distance/Å**				
An–O_NO_3__	2.514(2)	2.499(3)	2.492(2)	2.484(2)
	2.515(2)	2.511(4)	2.496(2)	2.497(2)
	2.518(2)	2.516(4)	2.500(2)	2.508(2)
	2.521(2)	2.524(4)	2.503(2)	2.508(2)
	2.523(2)	2.525(4)	2.505(2)	2.511(2)
	2.525(2)	2.531(4)	2.506(2)	2.516(2)
C=O	1.255(5)	1.268(6)	1.255(4)	1.271(3)
	1.261(5)	1.270(6)	1.260(4)	1.271(3)
C–N	1.303(5)	1.313(6)	1.318(4)	1.308(4)
	1.323(4)	1.314(6)	1.351(9)	1.311(3)

**H-bond param.** c				
D–H/Å	1.13	0.94	1.08	1.17
H⋯A/Å	1.28	1.48	1.34	1.24
D⋯A/Å	2.41	2.41	2.42	2.41
D–H⋯A/Å	178.3	167.9	178.0	177.1

a Ref. 16.

b This work.

c Notations related to hydrogen bonds. D: hydrogen bond donor, A: hydrogen bond acceptor.

Note that the coordination sphere of Np^4+^ is fully saturated by 12-coordination resulted from 6 bidentate NO_3_^−^, and that there are no direct interactions between Np^4+^ and L. Therefore, L does not play any roles as a ligand in both 3 and 4 despite its bridging nature that we found in 1-dimensional coordination polymers of U^VI^O_2_^2+^, [UO_2_(NO_3_)_2_(L)]_*n*_.^20,21^ Nevertheless, L still plays another important role to construct the crystal structures of 3 and 4. Negative charge of [Np(NO_3_)_6_]^2−^ has to be somehow compensated in crystal structures by incorporation of countercation(s). Based on the experimental conditions described above, no cations other than Np^4+^ and H^+^ were available in the current reaction systems. Therefore, the most plausible countercation in 3 and 4 should be H^+^. While usual status of H^+^ in aqueous solution is oxonium ion like H_3_O^+^, no isolated O atoms attributable to H_3_O^+^ have been found in the residual Fourier maps in both structures despite their deposition from 3 M HNO_3_ aq. Instead, a significant residual electron density was found between two carbonyl O atoms of neighbouring L molecules in both 3 and 4, and assigned to H^+^ with isotropic temperature factor. The final least-squares refinement of each structure was successfully converged to afford good agreement factors; *R* = 0.0192 (*I* > 2*σ*), w*R* = 0.0420 (all) for 3; *R* = 0.0188 (*I* > 2*σ*), w*R* = 0.0691 (all) for 4. Therefore, we conclude that anhydrous H^+^ is present as a countercation of [Np(NO_3_)_6_]^2−^ in both 3 and 4.

**Fig. 3:**
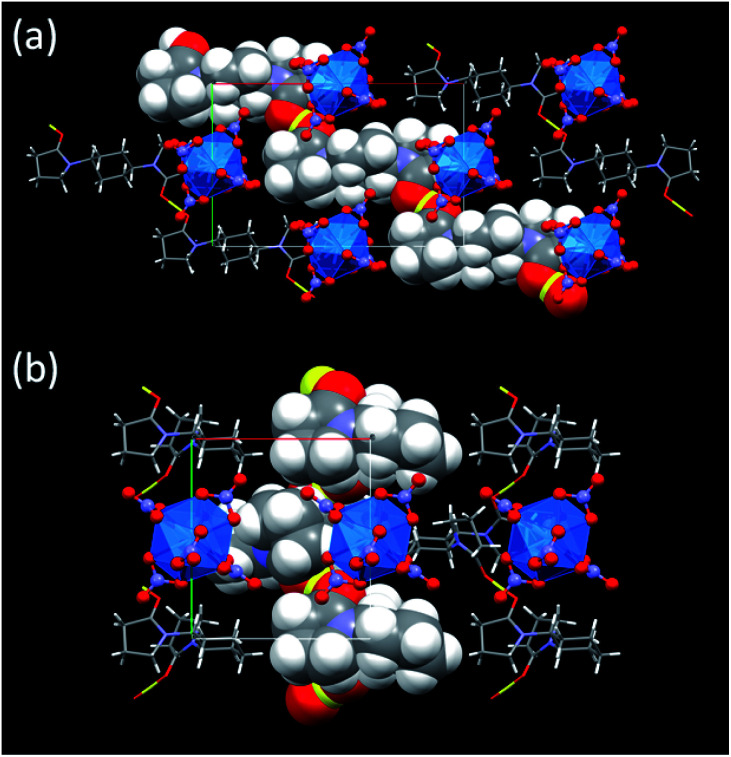
Crystal structures of (a) (HL1)_2_[Np(NO_3_)_6_] (3) and (b) (HL2)[Np(NO_3_)_6_] (4) along *c* axes. Yellow: H^+^, blue: Np, red: O, purple: N, grey: C, white: H. Blue transparent icosahedron shows coordination geometry around Np^4+^ centre surrounded by 6 bidentate NO_3_^−^. One of [H⋯L]_*n*_ hydrogen bond polymers in each structure is drawn in van der Waals radius for its clarity.

During crystallization process, initial hydration around H^+^ in the reaction mixtures was fully removed. Instead, the dehydrated H^+^ interacts with the neighbouring L molecules to make a [H^+^⋯L]_*n*_ hydrogen bond polymer as shown in Fig. 3. In 3, [H^+^⋯L1] units are extended through translational operation (1/2, −1/2, 0) to form the zigzag-type 1D hydrogen bond polymer. In 4, [H^+^⋯L2] units are expanded by 2_1_ screw rotation along *b* axis to make a helical hydrogen bond polymer. As racemic L2 was used here, both *z*- and *s*-twisted helixes arising from the (*S*,*S*)- and (*R*,*R*)-isomers of L2, respectively, occur in this crystal structure. According to a comprehensive review by Steiner,^32^ all the hydrogen bond parameters listed in Table 1 indicate that these interactions in 3 and 4 are strongly covalent (63–170 kJ mol^−1^). However, its strength is still much weaker than that of an actual O–H bond (*e.g.*, 436 kJ mol^−1^ for methanol).^33^ Indeed, the C=O groups (mean 1.26 Å for 3, mean 1.27 Å for 4) are slightly longer than free L (1.23 Å for both L1 and L2),^16^ but still exhibit double bonding character on the basis of covalent radii (C=O: 1.24 Å, C–O: 1.43 Å).^34^ This means that there is no direct protonation to make an actual covalent bond between the carbonyl O and H^+^.

Unlike characteristic olive colour of ordinary Np(iv) species, [Np(NO_3_)_6_]^2−^ in 3 and 4 do not exhibit any colour as shown in Fig. 1. At present, the solid-state UV-vis spectra of the Np(iv) compounds are not available due to strong radioactivity of Np samples. Quantitative discussion on light absorptivity at solid state would anyway be complicated due to difficulty in normalization of absorption intensity, as we previously experienced in a comparison between [U(NO_3_)_6_]^2−^ crystalline compounds with different colours.^16^ However, the photomicrographs shown in Fig. 1 obviously contain some information about visual colour of 3 and 4. To make our discussion more quantitative, each pixel of these photographs was divided into colour appearance parameters, hue, saturation, and brightness by ImageJ.^26^ Here, hue defines intrinsic colour of a subject of interest. By comparing hue value of the crystals in Fig. 1 with that of the background, it is possible to quantify how these Np(iv) compounds are coloured. Fig. S1 and S2 (ESI†) show 8 bit grey scale colour components of photomicrographs of 3 and 4, respectively. While distribution is somewhat broad due to cracks, boundaries, and surface roughness, hue histograms of the crystal surfaces of 3 and 4 exhibit similar maxima to those of the background in both systems. These results clearly indicate that both Np(iv) compounds reported here are indeed colourless.

This fact can be explained by the Laporte selection rule. In a centrosymmetric system of the current [Np(NO_3_)_6_]^2−^, the electronic transitions between energy states with the same parity like f–f transitions in 5f^3^ electronic configuration of Np^4+^ are strictly forbidden. Decolouration of Np compounds due to the same reason have also been observed in 1 : 2 complexes of Np^V^O_2_^+^ with diglycolamide,^35^ oxidiacetate,^36^ and dipicolinate,^37^ where the inversion centre is present at the centre metal, Np^5+^, with 5f^2^ configuration.^38^

In order to theoretically confirm that Np compounds synthesized in this work are truly colourless, the intensities of 5f–5f electronic transitions in the [Np(NO_3_)_6_]^2−^ entity of 3 has been estimated by CASSCF/sc-NEVPT2 approach using ORCA program.^27^ The calculated spectrum are given in Fig. S3 (ESI†) together with those of [Np(H_2_O)_*n*_]^4+^ (*n* = 8, 9)^28^ as references which exhibit typical olive green colour. The structures of [Np(H_2_O)_*n*_]^4+^ were optimized by DFT calculations^29^ at the B3LYP level in water. In CASSCF calculations, for all complexes, three electrons were distributed among seven active 5f orbitals (CAS(3,7)) and both quartet and doublet electronic configurations were considered for which 35 and 112 roots were included, respectively. Remind that only absorption stemming from 5f–5f transitions are included in these calculations and neither 5f–6d nor LMCT states are represented in the spectra. Furthermore, vibronic progression to the electronic transitions are not included here. As can be seen in Fig. S3,† typical absorption features of tetravalent Np are well-represented in the spectra of [Np(H_2_O)_8_]^4+^ and [Np(H_2_O)_9_]^4+^ which are characterized by two strong absorptions at around 500–600 nm and at around 900 nm. Transition energies are overall somewhat overestimated as we found previously in the case of U(iv) complexes. This is because of the use of isolated highly charged cation and thereby overestimating the effective charge on actinide centre.^16^ By contrast, these absorption features are totally diminished in the spectra of [Np(NO_3_)_6_]^2−^ unit of 3. Closer look into the entire scale of the spectra (150–5000 nm) reveals that all 5f–5f transitions are strictly forbidden in this complex due to the perfect centrosymmetry of the complex. In reality, there are vibronic progressions to the electronic transitions,^39^ which are not included in our calculations. These effects may eventually give rise to absorption. However, as was previously demonstrated in the case of [U(NO_3_)_6_]^2−^,^16^ slight distortion to the *T*_h_ symmetry of [An^IV^(NO_3_)_6_]^2−^ causes clear colorization of the complex. It suggests that symmetry plays by far the most crucial role to the colour of complexes compared to marginal contribution from vibrational progression. Therefore, we believe our calculations excluding vibrational progressions are still valid to discuss the colourless features of Np(iv) compounds.

The U(iv)-analogues 1 and 2 we reported previously do not exhibit typical green colour unlike ordinary U(iv) species, but are still pale colored.^16^ In contrast, the corresponding Np(iv) compounds 3 and 4 are more colourless as shown in Fig. 1 and as demonstrated by the colour component analysis. Interionic distances like H^+^⋯[An(NO_3_)_6_]^2−^ and [An(NO_3_)_6_]^2−^⋯[An(NO_3_)_6_]^2−^ and lattice parameters are almost identical between corresponding U(iv)- and Np(iv) compounds (Fig. S4, Tables S1 and S2, ESI†). Therefore, there are no large differences in electrostatic interactions that may cause perturbation of the centrosymmetric geometry of [An(NO_3_)_6_]^2−^. The only difference currently found is the decrease in the An–O_NO_3__ bond distances by ∼0.02 Å, arising from the actinide contraction. Thus, the more compact structure of [Np(NO_3_)_6_]^2−^ compared to [U(NO_3_)_6_]^2−^ would suppress the static and/or dynamic structural perturbation from the centrosymmetry, presumably making 3 and 4 more colourless.

## Conclusions

In conclusion, we have succeeded in crystallization of [Np(NO_3_)_6_]^2−^ with anhydrous H^+^ and double-headed 2-pyrrolidone derivatives, L1 and L2. The isomorphic and isostructural features of the hexanitrato complexes of U^4+^ and Np^4+^ clearly indicate that the crystal structure of (HL)_2_[An(NO_3_)_6_] is common in coordination chemistry of An(iv) regardless of electronic configurations in 5f orbitals. On the other hand, some minor differences arising from the actinide contraction have also been observed in bond distances and colour of compounds. To further explore the systematic trend in An^4+^ coordination chemistry, our next target is Pu^4+^, which has 5f^4^ electronic configuration and has the highest relevance among An^4+^ in the nuclear fuel recycling. To our best knowledge, isolation of anhydrous H^+^ from aqueous systems without any direct covalent bonds and formation of hydrogen bond polymer [H^+^⋯L]_*n*_ are also quite rare.^17^ We also intend to further explore these unique and interesting aspects of H^+^-involving chemical interactions constructed by linker molecules designed and optimized appropriately.

## Conflicts of interest

There are no conflicts to declare.

## Supplementary Material

RA-010-C9RA10090C-s001

RA-010-C9RA10090C-s002
