# Supplementary material for: Crystallization of colourless hexanitratoneptunate(iv) with anhydrous H+ countercations trapped in a hydrogen bonded polymer with diamide linkers
Source: RSC Adv. 2020 Feb 7;10(10):6082–7. doi: 10.1039/c9ra10090c (PMC9049546; doi:10.1039/c9ra10090c)
Supplement: RA-010-C9RA10090C-s001 [file RA-010-C9RA10090C-s001.pdf]

Electronic Supplementary Material (ESI) for RSC Advances.
This journal is © The Royal Society of Chemistry 2020

Electronic Supplementary Information

Crystallization of colourless hexanitoratoneptunate(IV) with anhydrous H^+ countercations trapped into hydrogen bond polymer with diamide linkers

Koichiro Takao,*,† Juliane März,‡ Moe Matsuoka,† Takanori Mashita,† Hiroyuki Kazama† and Satoru Tsushima†,‡,§

† Laboratory for Advanced Nuclear Energy, Institute of Innovative Research, Tokyo Institute of Technology, 2-12-1 N1-32, O-okayama, Meguro-ku, 152-8550 Tokyo Japan.

‡ Institute of Resource Ecology, Helmholtz-Zentrum Dresden-Rossendorf (HZDR), Bautzner Landstraße 400, 01328 Dresden, Germany.

§ Tokyo Tech World Research Hub Initiative (WRHI), Institute of Innovative Research, Tokyo Institute of Technology, 2-12-1, O-okayama, Meguro-ku, 152-8550 Tokyo, Japan.

	page
**Fig. S1.** Colour component analysis for (H**L1**)$_2$[Np(NO$_3$)$_6$] (**3**).	S2
**Fig. S2.** Colour component analysis for (H**L2**)$_2$[Np(NO$_3$)$_6$] (**4**).	S2
**Fig. S3.** Absorption spectrum of the $Np(NO_3)_6^{2-}$ unit in complex **3** together with those of $[Np(H_2O)_n]^{4+}$ (n = 8, 9) calculated at the CASSCF (3,7) level showing the electronic transitions stemming from the f-f transitions.	S3
**Fig. S4.** Schematic structures of $[Np(NO_3)_6]^{2-}$ and [H$^+$···**L**]$_n$ hydrogen bond polymer in (a) **3** (**L** = **L1**) and (b) **4** (**L** = **L2**).	S4
**Table S1.** Crystallographic data of (H**L1**)$_2$[An(NO$_3$)$_6$] (An = U, Np).	S5
**Table S2.** Crystallographic data of (H**L2**)$_2$[An(NO$_3$)$_6$] (An = U, Np).	S6
References.	S7

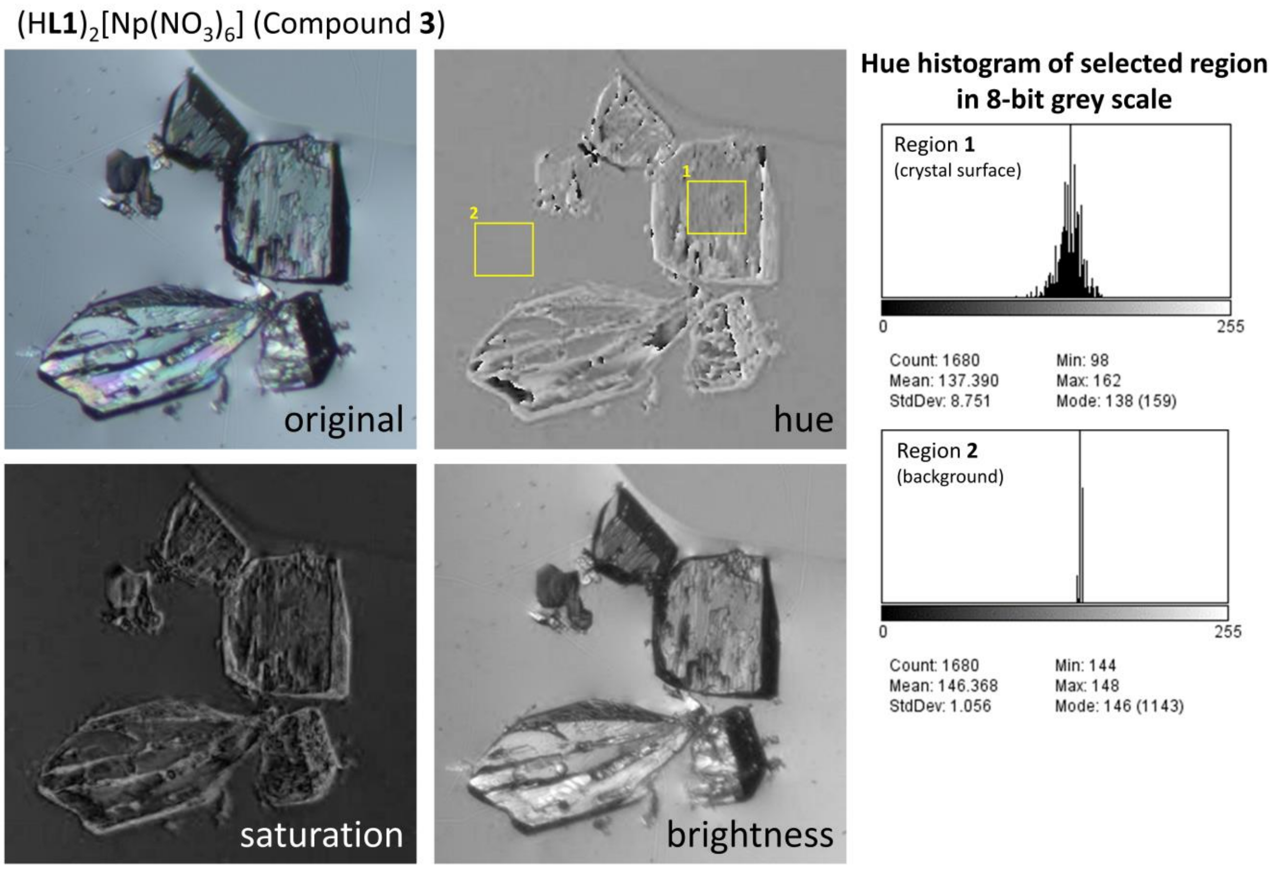

Fig. S1. Colour component analysis for (H**L1**)$_2$[Np(NO$_3$)$_6$] (**3**).

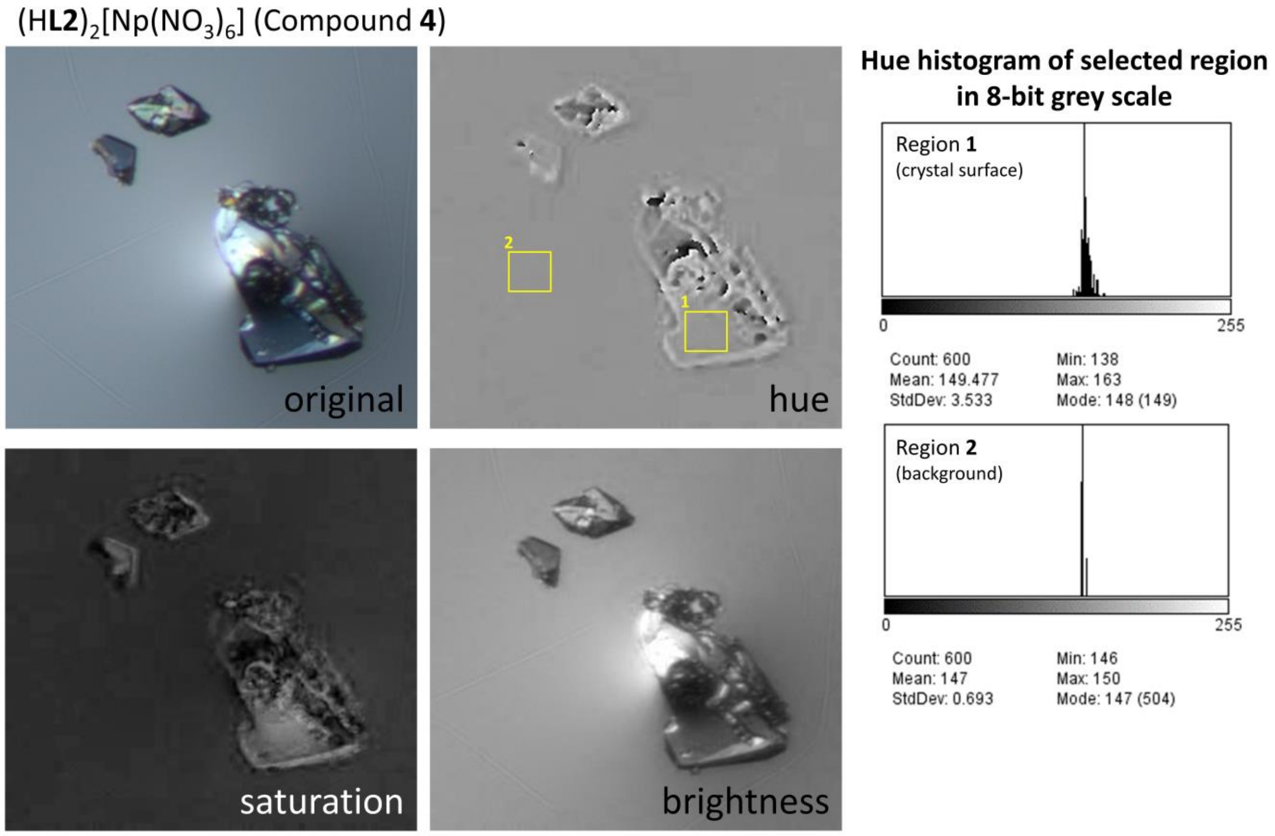

Fig. S2. Colour component analysis for (H**L2**)$_2$[Np(NO$_3$)$_6$] (**4**).

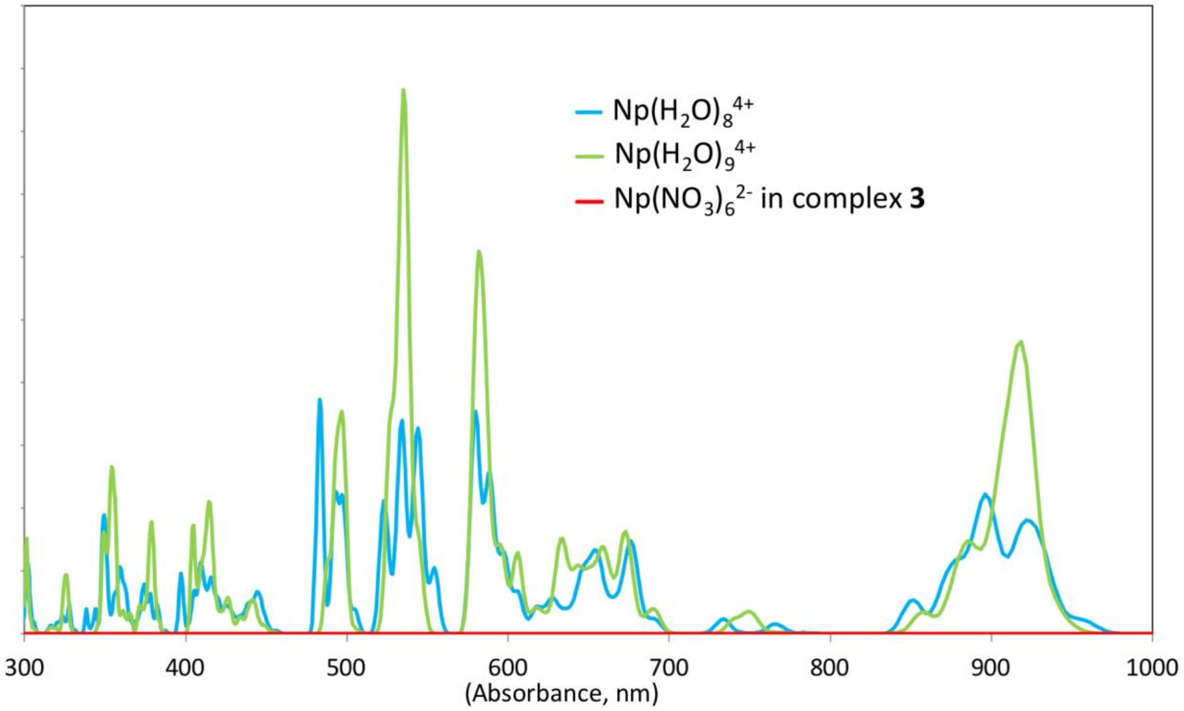

Fig. S3. Absorption spectrum of the $Np(NO_3)_6^{2-}$ unit in complex **3** together with those of $[Np(H_2O)_n]^{4+}$ (n = 8, 9) calculated at the CASSCF (3,7) level showing the electronic transitions stemming from the f-f transitions. For $[Np(H_2O)_n]^{4+}$ (n = 8, 9), corresponding structures were taken from those based on DFT calculations at the B3LYP level in water.

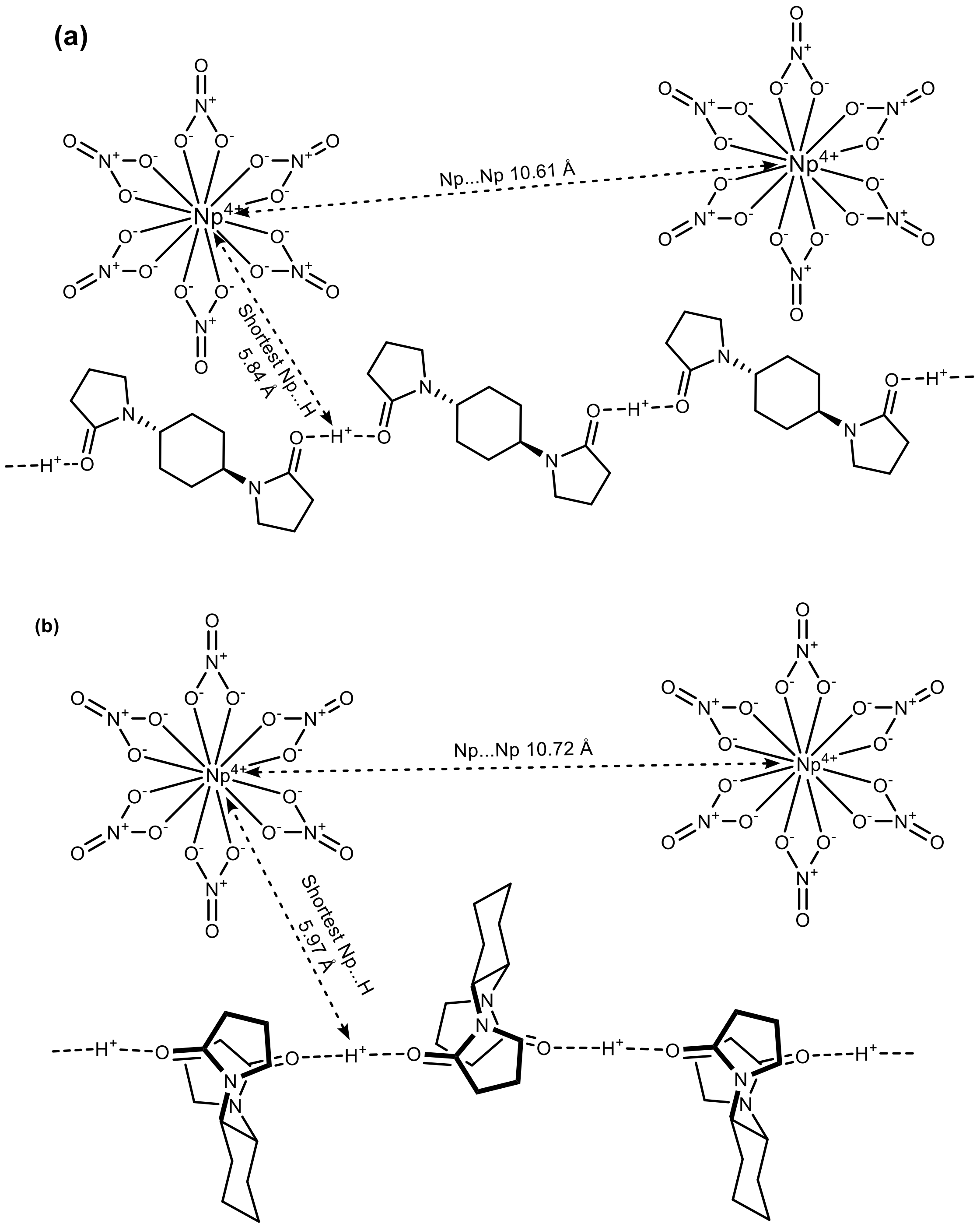

Fig. S4. Schematic structures of $[Np(NO_3)_6]^{2-}$ and $[H^+\cdots\mathbf{L}]_n$ hydrogen bond polymer in (a) **3** (**L** = **L1**) and (b) **4** (**L** = **L2**).

Table S1. Crystallographic data of $(HL1)_2[An(NO_3)_6]$ (An = Np, U).

	$(H\mathbf{L1})_2[Np(NO_3)_6]$ (**3**)[a]	$(H\mathbf{L1})_2[U(NO_3)_6]$ (**1**)[b]
formula	$C_{28}H_{46}N_{10}NpO_{22}$	$C_{28}H_{46}N_{10}O_{22}U$
formula weight	1113.76	1112.75
crystal size (mm)	0.075 × 0.098 × 0.157	0.414 × 0.300 × 0.286
crystal system	monoclinic	monoclinic
space group	*C*2/*c* (#15)	*C*2/*c* (#15)
a (Å)	18.1413(10)	18.1738(6)
b (Å)	10.9944(6)	11.0137(3)
c (Å)	21.6888(12)	21.8047(7)
β (°)	109.931(2)	109.958(8)
V (Å^3)	4066.8(4)	4102.3(3)
Z	4	4
T (K)	100	123
D_{calcd} (g·cm^{-3})	1.814	1.802
μ (mm^{-1})	2.652	4.054
obsd data (all)	4312	4696
R ($I > 2\sigma$)	0.0192	0.0228
wR (all)	0.0420	0.0549
GOF	1.071	1.082
$\Delta\rho_{max}$ (e$^-$·Å^{-3})	0.474	1.03
$\Delta\rho_{min}$ (e$^-$·Å^{-3})	−0.523	−0.36

[a] This work. [b] Ref. S1

Table S2. Crystallographic data of (H**L2**)$_2$[An(NO$_3$)$_6$] (An = U, Np).

	(H**L2**)$_2$[Np(NO$_3$)$_6$] (**4**)[a]	(H**L2**)$_2$[U(NO$_3$)$_6$] (**2**)[b]
formula	$C_{28}H_{46}N_{10}NpO_{22}$	$C_{28}H_{46}N_{10}O_{22}U$
formula weight	1113.76	1112.75
crystal size (mm)	0.080 × 0.098 × 0.110	0.335 × 0.276 × 0.266
crystal system	monoclinic	monoclinic
space group	$P2_1/n$ (#14)	$P2_1/n$ (#14)
a (Å)	9.8264(10)	9.8222(4)
b (Å)	10.7164(11)	10.7334(4)
c (Å)	19.517(2)	19.5516(8)
β (°)	103.175(4)	103.185(7)
V (Å^3)	2001.1(4)	2006.89(15)
Z	2	2
T (K)	100	123
D_{calcd} (g·cm^{-3})	1.848	1.841
μ (mm^{-1})	2.695	4.143
obsd data (all)	4249	4580
R ($I > 2\sigma$)	0.0188	0.0433
wR (all)	0.0691	0.0821
GOF	1.340	1.078
$\Delta\rho_{max}$ (e$^-$·Å^{-3})	0.975	2.36
$\Delta\rho_{min}$ (e$^-$·Å^{-3})	−1.196	−2.20

[a] This work. [b] Ref. S1

References.

S1. Takao, K.; Kazama, H.; Ikeda, Y.; Tsushima, S., Crystal Structure of Regularly T_h -Symmetric $[U(NO_3)_6]^{2-}$ Salts with Hydrogen Bond Polymers of Diamide Building Blocks. *Angew. Chem. Int. Ed.* **2019,** *58,* 240-243.
